# Origin of the inhomogeneous nanoscale resistivity in chromium doped V_2_O_3_

**DOI:** 10.1038/s41598-025-99892-y

**Published:** 2025-05-06

**Authors:** Johannes Mohr, Yudi Wang, Xiaoyu Xu, Ruilin Wang, Dirk J. Wouters, Rainer Waser, Joyeeta Nag, Daniel Bedau

**Affiliations:** 1 Institute of Materials in Electrical Engineering and Information Technology II, RWTH Aachen University, 52074 Aachen, Germany; 2 Western Digital San Jose Research Center, 5601 Great Oaks Parkway, San Jose, CA 95119 USA; 3 Research Center Jülich, Peter Grünberg Institute PGI 7, 52428 Jülich, Germany

**Keywords:** Doped V_2_O_3_, Grain boundaries, Conductive AFM, Mott-insulators, Electronic devices, Electronic properties and materials

## Abstract

Chromium doped V_2_O_3_ polycrystalline thin films typically consist of conductive grains separated by insulating grain boundaries. We investigate the origin of the spatially inhomogeneous resistivity in these films and find no qualitative differences between doped and undoped films, or for different oxygen stoichiometries. By a combination of conductive atomic force microscopy, high-resolution transmission electron microscopy and nanoscale elemental mapping, we show that the inhomogeneity is due to the formation of amorphous or poorly crystallized regions at the grain boundaries. Both the distribution of the dopants and the local oxygen stoichiometry appear to be very homogeneous, and therefore do not contribute to the inhomogeneous conductivity.

**Supplementary Information:**

The online version contains supplementary material available at 10.1038/s41598-025-99892-y.

## Introduction

Chromium doped V_2_O_3_ has attracted significant attention due to the complicated correlated electron physics at play in this material. Recent investigations have demonstrated an electrical threshold switching effect^1^, which was shown to be very promising for constructing selector elements for dense crossbar memories^2,3^, as well as for neuromorphic computing^4^. However, one major challenge is translating these properties from fundamental research on single crystals or epitaxially grown films to practical, integrated applications based on polycrystalline V_2_O_3_ films. Here, a significant influence of the grain boundaries on the macroscopic properties can be expected, more so as we move to ever smaller structures in microelectronics. While it has been shown recently that the Mott-transition can be observed even in very thin, polycrystalline films^5^, it has also been shown that such films exhibit a substantially inhomogeneous conductivity on the nanoscale^5,6^, which appears to be correlated to the grain structure. Such inhomogeneity has important implications for small devices and might impose an ultimate scaling limit due to the induced device-to-device variability. Consequently, it is critical to understand the origin of this inhomogeneity and to determine whether it is an intrinsic material limitation, or an engineering challenge that can be mitigated by controlling the right process parameters.

To identify the potential causes of the observed inhomogeneous conductivity we employ a correlative microscopy approach, combining conductive atomic force microscopy (c-AFM), to map the local resistivity distribution, with transmission electron microscopy (TEM) to examine the material’s local structure and composition. While previous studies have consistently shown that the films consist of relatively conductive grains separated by insulating grain boundaries, different explanations have been considered: The first hypothesis is a segregation of chromium at the grain boundaries; it is well known that higher doping concentrations render the films more resistive^6–9^, and the doping concentrations typically employed in devices are quite large^2^, so this explanation appears plausible. However, as we will demonstrate, there is strong evidence against this explanation^5^. The second hypothesis is a difference in the oxygen stoichiometry between the interior of the grains and the grain boundaries, which might occur as the diffusion coefficient of oxygen at the surface could be higher than in the bulk. Finally, V_2_O_3_ is also very sensitive to strain, which might differ spatially due to the growth condictions^10^.

Our results show that the conductivity variations are not caused by a segregation of chromium at the grain boundaries or differences in oxygen stoichiometry. Instead, we observed that the insulating nature of the grain boundaries is due to the formation of amorphous or poorly crystallized regions.

## Results and discussion

All Cr:V_2_O_3_ films were deposited using reactive radio-frequency (RF) magnetron sputter deposition from metallic targets in an oxygen containing atmosphere. The targets used were either pure vanadium or a vanadium/chromium alloy containing 15% Cr. A total gas flow of 100 sccm was used, consisting of a mixture of pure argon, and a mixture of 1% O_2_ in Ar. This was done because the required oxygen partial pressure to fabricate V_2_O_3_ is very low, otherwise higher oxides such as VO_2_ and V_2_O_5_ are formed. The substrates were placed on a rotating holder which was heated by halogen bulbs on the backside to a deposition temperature of 600 °C. Using X-ray diffraction, it was shown that this results in the crystallization of the correct phase^6^.

For the c-AFM measurements, the films were deposited on oxidized silicon wafers covered with a 5 nm Ti adhesion layer and a 30 nm Pt bottom electrode. Coupons were mounted to AFM sample discs with conductive silver paint which was also used to make an electrical connection to the bottom electrode. The samples were stored under an inert argon or nitrogen atmosphere immediately after deposition and between measurements to prevent absorption of ambient moisture into the films as much as possible, as well as a contamination of the surface, which might influence the measured resistivities. However, it should be noted that a very thin moisture layer on top of the films will normally be present in any c-AFM performed under ambient atmosphere^11–13^.

A full factorial experiment design was employed, considering as potential influences the doping concentration (undoped or 15% chromium doped), the oxygen concentration (600 ppm or 1400 ppm oxygen content) and the film thickness (20 nm or 60 nm). The latter was chosen as a proxy for other properties that cannot directly be tuned, such as the grain size and morphology. The resulting eight different samples are given in Table 1.

**Table 1:** The samples characterized with c-AFM.

Sample	1	2	3	4	5	6	7	8
Cr doping (%)	15	15	15	15	0	0	0	0
Thickness (nm)	20	20	60	60	20	20	60	60
O_2_ (ppm)	600	1400	600	1400	600	1400	600	1400

For the TEM experiments, a film was deposited on a silicon nitride membrane, as this allows taking images perpendicular to the plane of the films, and thus acquire data that can directly be correlated to the c-AFM measurements. For high resolution imaging, very thin 5 nm SiN membranes were used with 10 nm Cr:V_2_O_3_ films. Unfortunately, the experimental effort of the TEM investigation did not permit imaging multiple films, therefore those conditions most applicable to devices were selected, which are 15% doping and 600 ppm oxygen^2,3^.

**Fig. 1:**
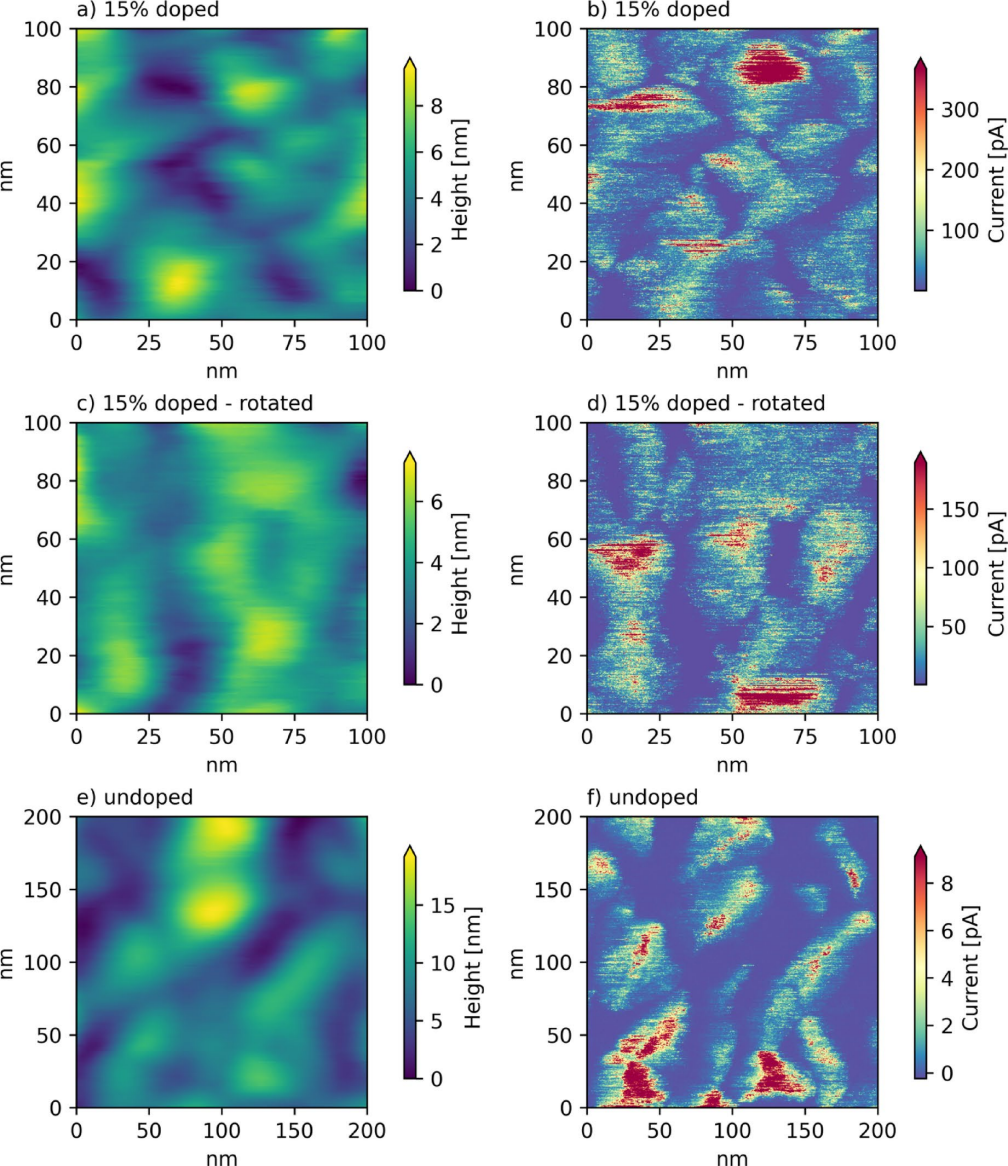
C-AFM measurements of doped and undoped V_2_O_3_. The left column shows the topography, the right one the measured current. Because different bias voltages had to be used, the absolute current values are not comparable between doped and undoped sample. The images (**c**) and (**d**) were taken on the same film as (**a**) and (**b**), but with the sample rotated in its plane by 90°.

As expected, c-AFM measurements reveal significant fluctuations in the local conductivity distribution, as shown in Fig. 1, which also confirms that the films consist of conductive grains separated by insulating grain boundaries. The measurements for all samples of the systematic investigation are shown in the supplementary Figure S1 and Figure S2. An important result is that the morphology and conductivity distribution are only weakly influenced by the doping concentration and oxygen flow. All films consist of approximately globular grains with diameters on the order of 20 nm to 100 nm, depending on the material. Addition of chromium doping seems to lead to a decrease in grain size; the oxygen flow has a negligible influence, with only a potential slight elongation of grain shapes observed at 1400 ppm oxygen. The conductivity distribution does not appear to show any qualitative differences, other than those corresponding to the changed grain structure. This is also the case for the influence of the film thickness, which appears to be limited to an increase in grain size for thicker films, accompanied by larger conductive domains. While the globular objects have so far been referred to as grains, this terminology needs to be used carefully, because it was not a-priori clear whether they are in fact single crystalline domains. Clarifying this was another key objective of the TEM study. Finally, caution is advised when interpreting the observed values of the current as resistivities, even though the applied voltage was the same for all eight measurements. While as expected, the average currents for the doped films are one to two orders of magnitude smaller, they are larger for the thick films than for thin ones, which would mean that the thick films exhibit a lower resistivity. This is unlikely, as the bottom 20 nm of the 60 nm films should not be too different from the 20 nm standalone films. Instead, it must be stressed that the measured current, even at a fixed voltage, is not a direct representation of the materials’ resistivity, because it also depends strongly on the contact area of the tip with the sample. This could very well be larger for the rougher topography of the 60 nm films. The influence of the contact area is even more severe for the high-resolution images in Fig. 1. For these, the bias voltage was tuned for the best imaging performance.

**Fig. 2:**
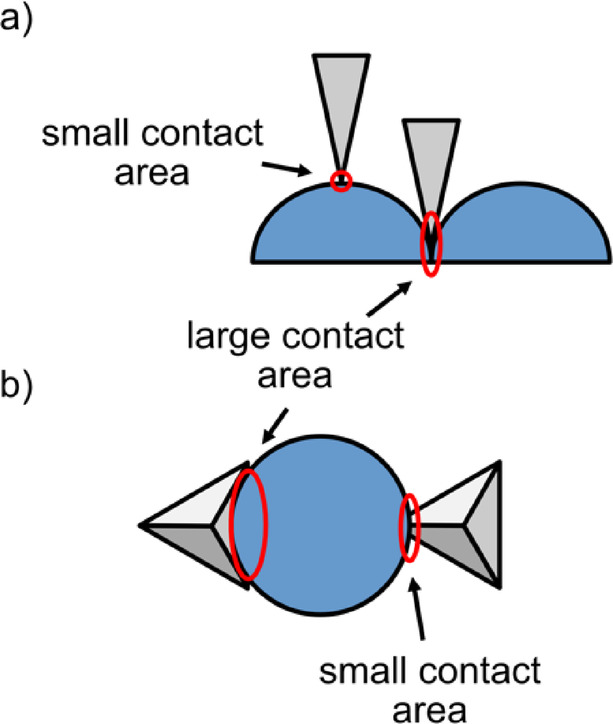
Contact areas between film and AFM tip for different geometries. (**a**) Viewed from the side. The contact area will be larger when the tip (grey) is in the depression between two grains (blue), because its sides also make contact. This results in a larger current. (**b**) Viewed from the bottom. The tip will have a larger or smaller contact area depending on which side of a grain it approaches.

It might appear that in addition to the insulating grain boundaries, there is indeed a conductivity fluctuation within the grains, with some spots showing much higher current levels. However, upon closer inspection it seems like the high-conductivity spots in Fig. 1b are always on the “north” faces of the crystallites. Of course, it is possible that some crystallographic directions are more conductive, but a simple experiment reveals that this is not the case. In Fig. 1c and d the same sample is shown, but rotated 90° in the AFM. Clearly, the conductive faces are still at the “north” face, demonstrating that this is an artifact from the AFM tip, which is not symmetric. This is illustrated in Fig. 2b: A non-symmetric tip can have different contact areas to a globular grain, depending on which side it makes contact to. If these spots are then ignored, it appears that the conductivity within the grains is approximately homogeneous. The tip contact area does not influence the result that the grain boundaries are insulating, because these are the lowest points of the surface topography and the contact area is therefore much larger than in the center of the grains, so there is a tendency to underestimate the resistivity (Fig. 2a).

These results are also a strong indication that the high resistivity of the grain boundaries is not due to a segregation of chromium, because the effect is just as pronounced in the completely undoped film shown in Fig. 1 e and f. Another possibility is a different stoichiometry at the grain boundaries, for example, they might be more permeable to oxygen and therefore more strongly oxidized. This explanation is unlikely because it is known that oxygen excess tends to make V_2_O_3_ more conductive^14^ in an effect qualitatively similar to Ti doping.^15^ If the oxidation was sufficiently strong that a completely different phase of vanadium oxide was formed this could explain the high resistivity, but such an effect should be even more pronounced at the top surface of the film, and thus a high resistivity should be measured everywhere. This is clearly not the case, giving a good indication that the origin might be rooted in local differences in the crystal structure of the material.

**Fig. 3:**
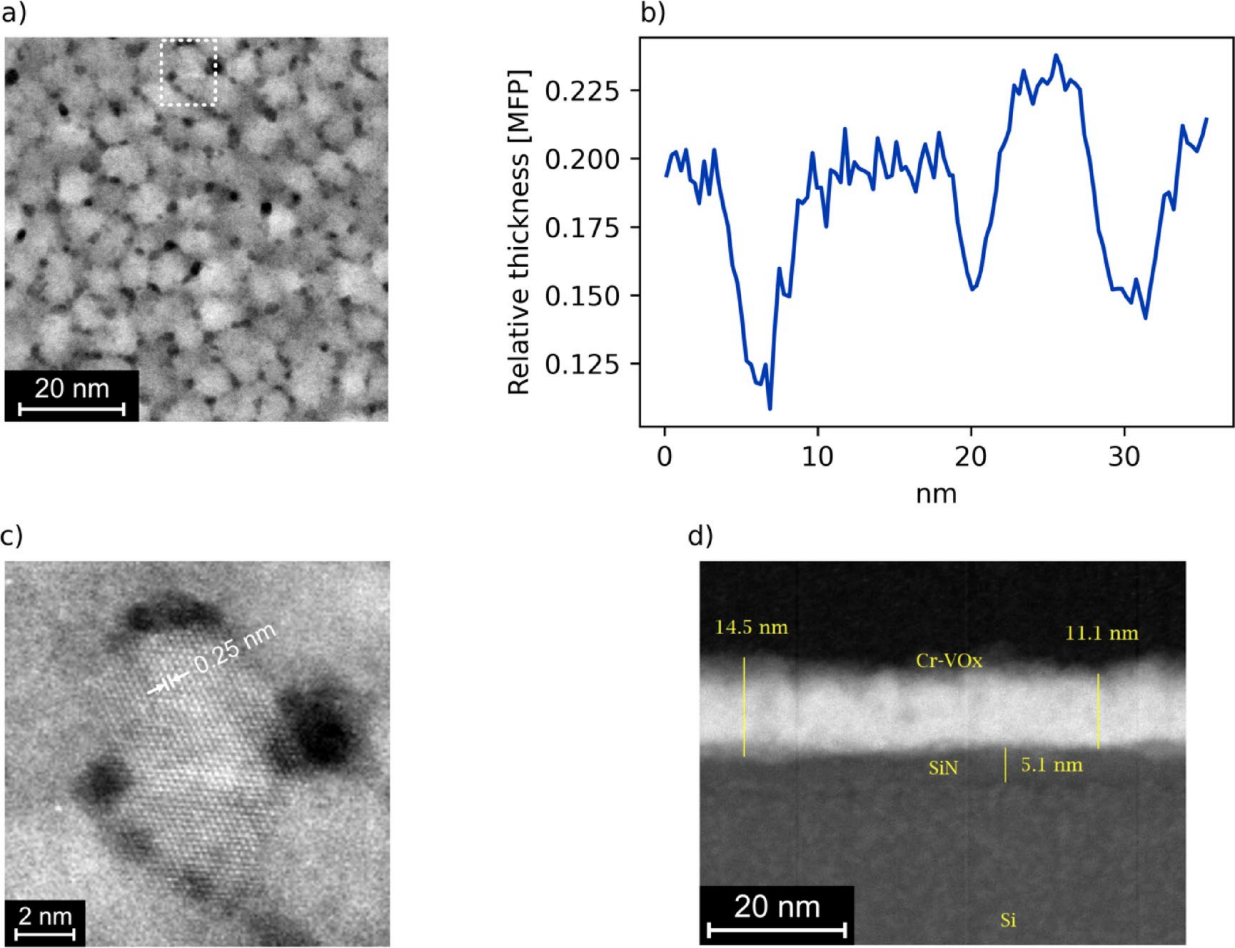
(**a**) Dark-field STEM image of the Cr:V_2_O_3_ film grown on the membrane. The dashed rectangle highlights a grain that is magnified in (**c**). Here, the crystal lattice is clearly visible. Note the hexagonal symmetry, indicating that this grain is oriented with the c-axis perpendicular to the surface. (**b**) Relative thickness in units of the inelastic mean free path (MFP) of the electrons. (**d**) A cross sectional view of the membrane and the Cr:V_2_O_3_ film. This section was prepared from the frame of the membrane, because of this, the supporting silicon substrate is also visible.

To confirm these conclusions, the TEM investigation proved helpful. The films grown on SiN membranes show a qualitatively similar growth mode to the c-AFM samples, consisting of small, globular grains, as seen in Fig. 3a. The grains are slightly smaller than those observed by AFM, which is most likely due to the thinner films used here. It might be suspected that this leads to a different structure of the grain boundaries, especially because they are grown on SiN and Pt, respectively. However, previous results indicate that in either case a columnar growth mode occurs, and because the 10 nm thickness of the layer is well above the initial nucleation phase, we believe the results to be comparable. The cross-sectional image in Fig. 3d was taken on a FIB lamella cut from the silicon frame of the membrane. The membrane thickness is confirmed to be 5 nm, the Cr:V_2_O_3_ film appears to be close to its target thickness of 10 nm, but with a significant variability. Details of the structure are not visible here, as it was not possible to prepare a thinner lamella than the 5–8 nm size of a single grain. Because of that, a membrane was chosen for high-resolution imaging. Again, in Fig. 3a, the grains seem to be separated by trenches at the grain boundaries, with some spots even appearing like holes. This is also shown by Fig. 3b. There is a much weaker scattering of the electrons at the grain boundaries, indicating that these areas are thinner. Of course, the inelastic MFP could also be influenced by the density and crystal structure. However, the two influences should largely compensate: An amorphous structure should lead to stronger scattering due to the disorder, but the lower density implies fewer scattering centers. On the other hand, in the crystalline material, there are more atoms (per volume) to scatter the electrons, yet the ordered structure means less frequent scattering events. Finally, Fig. 3c shows that the macroscopic grains in fact correspond to single crystalline domains, with the V_2_O_3_ lattice clearly visible. This lattice seems to extend precisely to the interfaces to other grains. Note also the hexagonal symmetry, which indicates that this grain has its c-axis perpendicular to the surface. The observed spacing of ~ 0.25 nm is consistent with the (110) planes of V_2_O_3_. This image also suggests that the grain boundaries are not coherent.

**Fig. 4:**
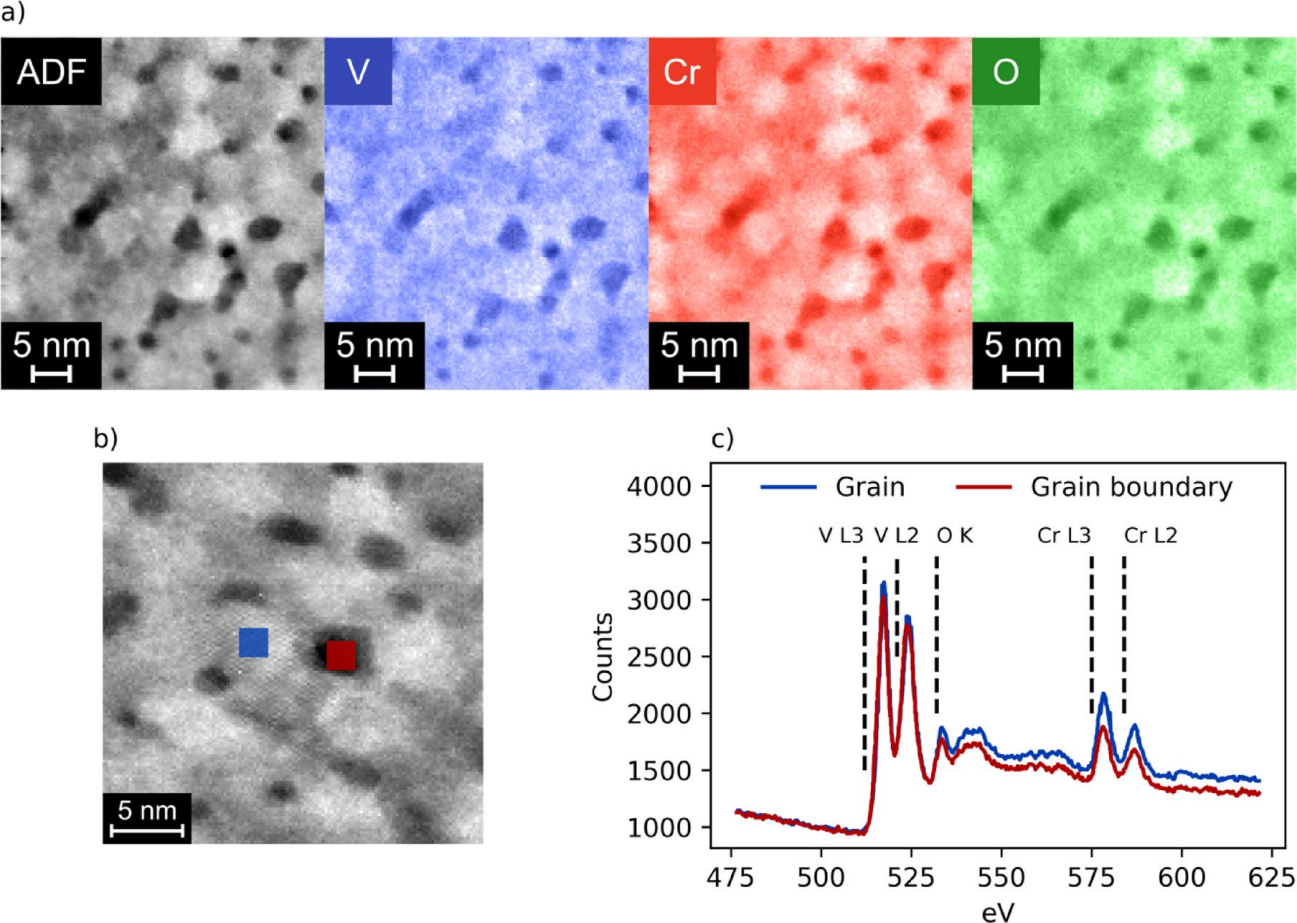
(**a**) Maps of the elemental distributions along with an annular dark-field (ADF) image of the film. (**b**) Regions within a grain (blue) and at a grain boundary (red) where EELS spectra were acquired. These are shown in (**c**).

To confirm that the differences in conduction are not a result of differences in composition, elemental maps were recorded. These are shown in Fig. 4a. The distributions of vanadium, chromium, and oxygen closely resemble each other and align with the topographic annular dark-field image. This clearly confirms that there is no significant segregation of chromium even for 15% doping concentration, and that the grain boundaries are not more oxidized compared to the interior of the grains. In Figure S3, the ratios V/Cr and (V + Cr)/O are shown, which appear to agree with this, and indicate only a minor excess of V at the grain boundaries. This finding is further supported by the EELS spectra shown in Fig. 4c, which were recorded for both a grain and a grain boundary, as indicated in Fig. 4b. They look nearly identical even with regard to minor features, indicating that the composition is the same. Additionally, although it is less pronounced here than in Fig. 3c, the crystal lattice is uniform within the grain and extends to its boundary, where it ceases to be visible. This confirms that the grain is single crystalline. The orientation of the crystallites in other grains must be sufficiently different so that the lattice is not seen there with the present orientation of the sample, as expected for a polycrystalline film.

**Fig. 5:**
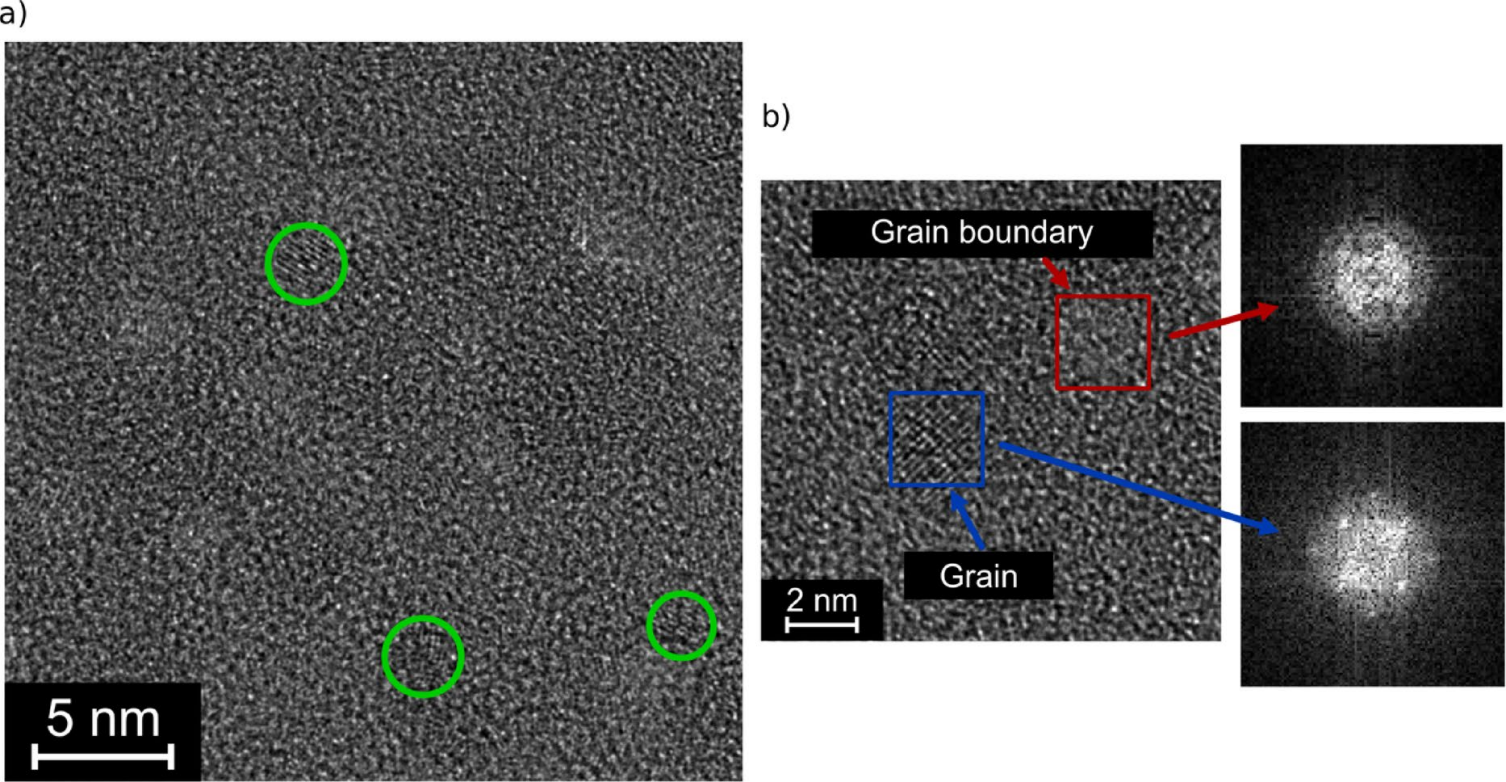
(**a**) High-resolution TEM (HR-TEM) image of the film. Some crystallites are highlighted in green. (**b**) Higher magnified HR-TEM of a grain and a grain boundary, with Fourier transforms of both areas.

The previous observations rule out doping and oxygen stoichiometry as the origin of the insulating grain boundaries, leaving as a likely cause a strained or poor-quality crystal lattice. To confirm this, high-resolution TEM images were taken. One of these is shown in Fig. 5a.

In multiple locations the lattice is clearly visible, with different orientations of the crystallites. This confirms the expected polycrystalline growth mode. In a previous study it was found that there is a certain preferential orientation of the c-axis out of the plane of the film,^5^ this does not appear to have a strong effect on this image. However, it must be considered that the crystallite orientation is quite sensitive to the growth conditions and might be different for a SiN substrate. Interestingly, certain areas do not appear crystalline, which seem to be predominantly located at the brighter areas. These would correspond to the thinner regions of the film, and thus the grain boundaries. It must however be considered that there is also an influence of the amorphous SiN membrane, giving the overall impression of small crystallites in an amorphous matrix. These crystallites correspond to the grains in Fig. 3a, whereas the amorphous regions likely are the dark spots there. In Fig. 5b a magnified view is shown, with one region clearly crystalline, which is also confirmed by spots in the FFT. These are somewhat faint due to the very small size of the crystallites in 10 nm films, but clearly absent from the region corresponding to a grain boundary. Therefore, it can be concluded that the boundary region is amorphous, or at least substantially less crystalline than the grain. Similar results were also obtained in different locations of the sample, confirming that this is a general trend. As it has been shown before that the resistivity of amorphous Cr:V_2_O_3_ is orders of magnitude higher than in the crystalline case^6,9^, it would be expected that these areas are found to be insulating in the c-AFM, which is consistent with the observations. Another indication that the material might contain amorphous regions is that while the films produced a clear diffracted signal in grazing incidence X-ray diffraction (XRD), this signal is very weak, with the diffraction pattern for a 10 nm film barely distinguishable from the background in a benchtop XRD.^5^ This could be due to a reduced diffracting volume compared to a fully crystallized film because of the amorphous domains.

## Conclusion

The presented evidence clearly shows that the insulating nature of the grain boundaries in polycrystalline (Cr):V_2_O_3_ thin films arises from the formation of amorphous or poorly crystallized regions, not from a segregation of dopants, from variations in stoichiometry, or the formation of higher valence oxides. This finding has significant implications, both fundamentally and for device applications. The homogeneous distribution of the dopants seems to indicate that the qualitative physical behavior should be the same for both low and high doping concentrations. Furthermore, in devices, typically high doping concentrations such as 15% are beneficial,^2^ and our work demonstrates that it might be possible to increase them even further, without promoting an additional conduction inhomogeneity. The absence of oxidation at the grain boundaries is promising because it indicates that a brief exposure of the film to the ambient atmosphere during processing will not have a detrimental influence. However, the amorphization of the grain boundaries presents a potential challenge because it might limit device scaling. This requires further research to clarify its origin.

Considering how well the results shown here will carry over to polycrystalline Cr:V_2_O_3_ films fabricated elsewhere and by other methods is somewhat challenging due to the very limited number of studies on them. Previous results on undoped V_2_O_3_ indicate that even when the film is produced by annealing an amorphous film sputtered at room temperature with a buffer couple to crystallize it^16^, the general pattern of insulating grain boundaries remains^6^. The insulating areas however appear smaller, which is consistent with a better crystalline quality due to the longer anneal and slower cooling. In addition, the observed currents are higher, as expected from macroscopic measurements of the conductivity of this material. On the other hand, this also leads to a significantly different morphology, which also strongly depends on the buffer couple used^16^. The grain structure of chromium doped films deposited by reactive co-sputtering appears quite similar to our films, with columnar grains separated by trench-like grain boundaries that occasionally even appear like pores^17^, indicating that at least qualitatively the results might transfer.

## Methods

### Sample fabrication

For the fabrication of the samples for the c-AFM measurements, oxidized silicon wafers that had previously been coated with a 5 nm Ti adhesion layer and 30 nm Pt were cut into 1″ × 1″ pieces. These were then cleaned by an ultrasonic treatment in acetone and isopropanol for 10 min each, they were then rinsed with deionized water and blown dry with nitrogen. The coupons were immediately introduced into the vacuum system. For the deposition, the coupons were heated to 600 °C, on reaching this temperature, a first 3-min pre-sputtering step behind a closed shutter was used to clean the target surface. The main deposition step was then started, with a power of 50 W applied to the 1″ targets, which consisted of either pure vanadium, or an 85% vanadium and 15% chromium alloy. The sputter sources were facing the samples approximately at a 45° angle. The pressure during deposition was controlled by an outflow valve to be 0.010 mbar. After the deposition was complete, the samples cooled in the chamber to a temperature of 100 °C before they were removed from the system. Any series of actual depositions was preceded by a dummy run to precondition the chamber. The deposition on the SiN membrane was done identically, except that a pre-cleaned membrane was used without initial cleaning steps to avoid any damage to the membrane from the sonication.

### Conductive AFM

The measurements were conducted with a Park Systems NX-10 AFM. Initially, the surface morphology was characterized using non-contact AFM with OMCL-AC160TS cantilevers. Conductive AFM measurements were performed with AS-2.8-SS conductive diamond cantilevers, which have a specified tip radius < 5 nm. For the larger area images in Figure S2, a common bias voltage of 0.1 V was applied to the sample holder, for the high-resolution images the voltage was tuned for each measurement; especially the undoped samples required much lower and negative voltages for stable imaging. It was verified that the topography measured during the c-AFM qualitatively matched the one observed in the non-contact measurements to verify that no damage was caused to the sample, and that the tip did not degrade from contact with the surface.

### TEM

The cross-sectional sample for TEM analyses was prepared using a Helios 600 Focused Ion Beam (FIB). A protective carbon film was deposited inside the FIB on top of the film at the frame of the membrane prior to the sample preparation. A JEOL ARM-200CF electron microscope equipped with Gatan Quantum 965 ER imaging filter (GIF) was used for TEM analysis.

## Electronic supplementary material

Below is the link to the electronic supplementary material.

Supplementary Material 1.

## Data Availability

The data that support the findings of this study are available from the corresponding authors upon reasonable request.
