# Supplementary material for: Origin of the inhomogeneous nanoscale resistivity in chromium doped V2O3
Source: Sci Rep. 2025 May 6;15:15826. doi: 10.1038/s41598-025-99892-y (PMC12056188; doi:10.1038/s41598-025-99892-y)
Supplement: Supplementary file 1 — Supplementary Material 1. [file 41598_2025_99892_MOESM1_ESM.pdf]

Supplementary Material

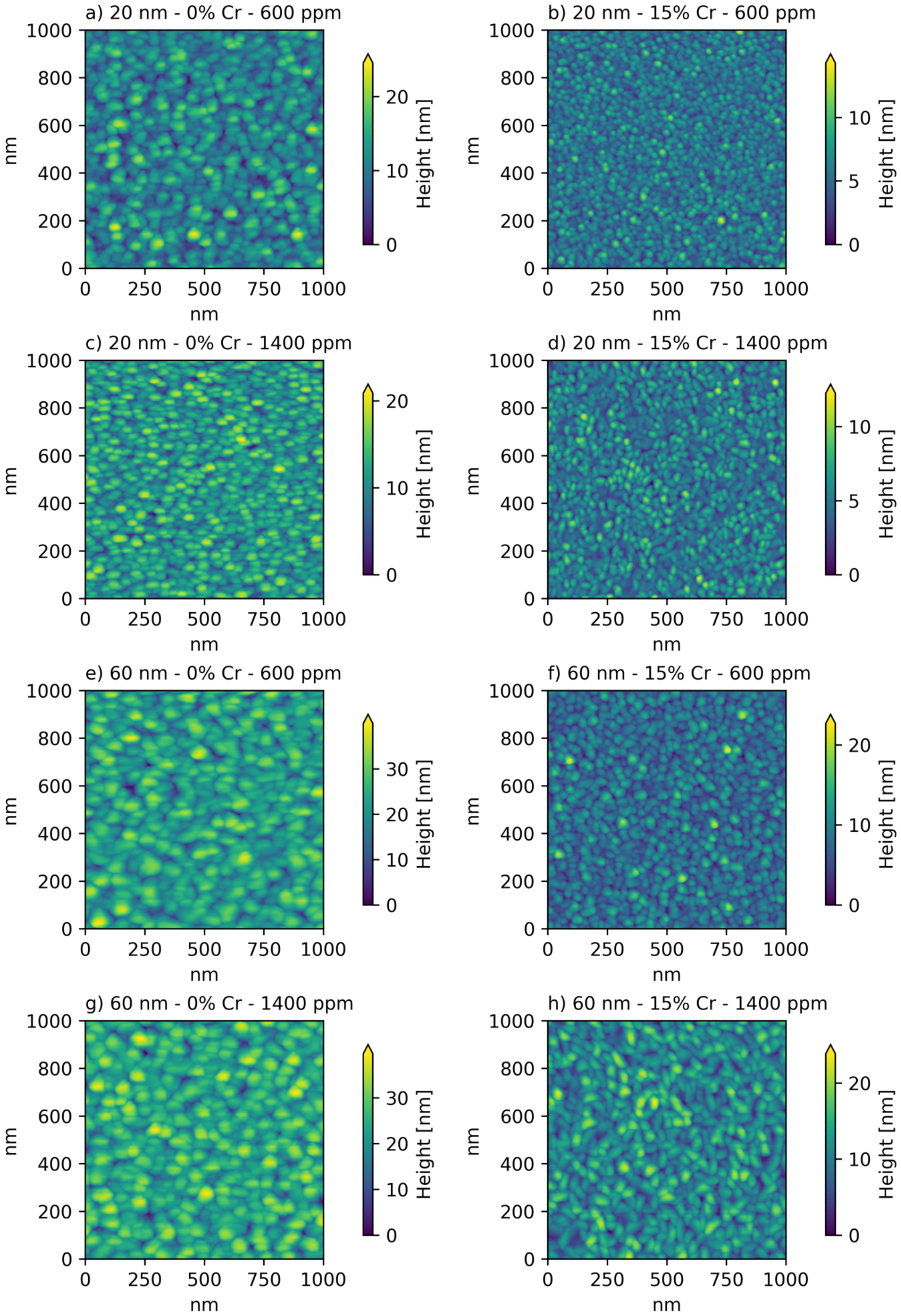

Figure S1: Non-contact topography measurements of 1 µm × 1 µm areas on all eight samples.

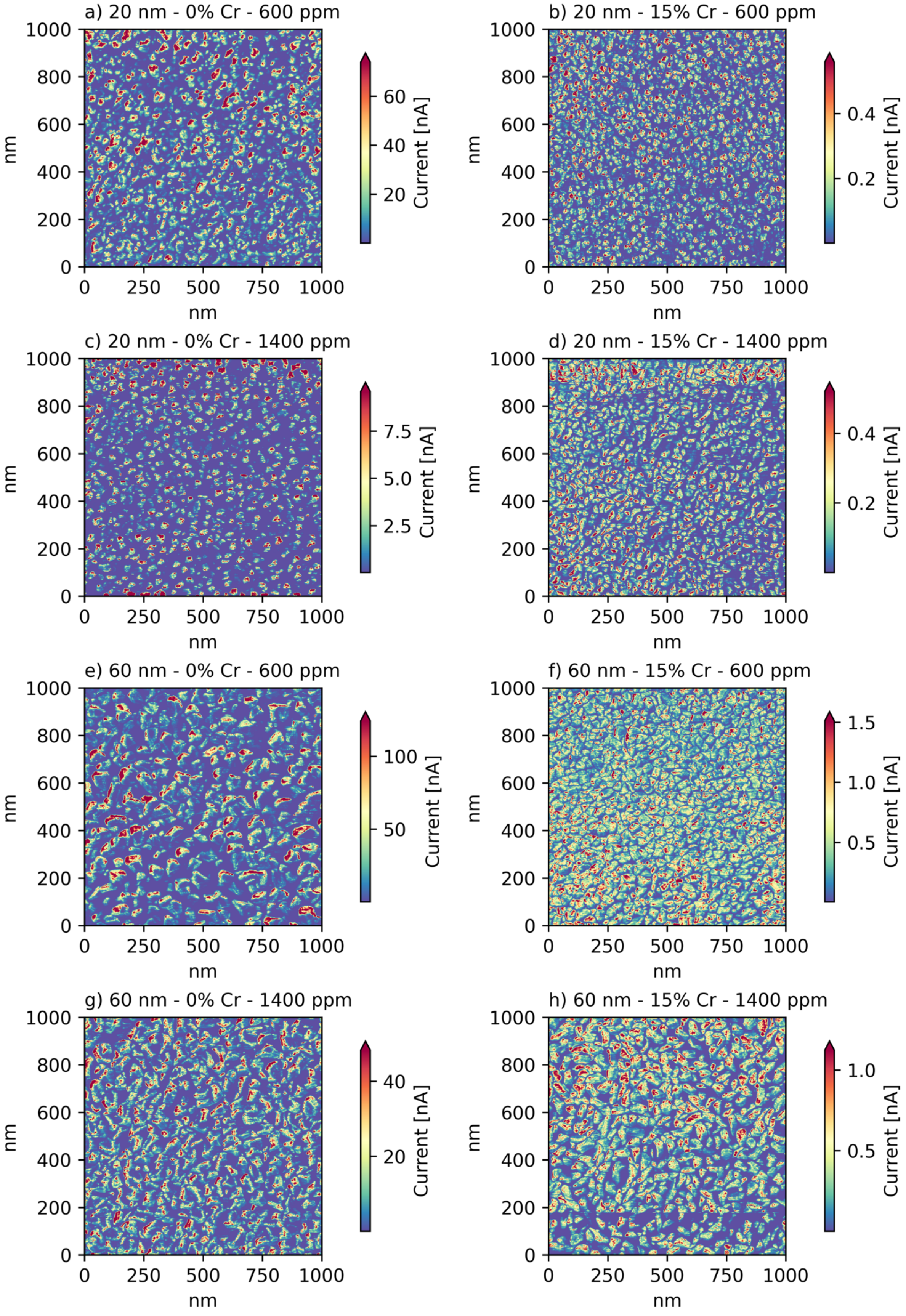

Figure S2: Local current measurements of 1 µm × 1 µm areas on all eight samples. A bias voltage of 0.1 V was applied to the sample holder.

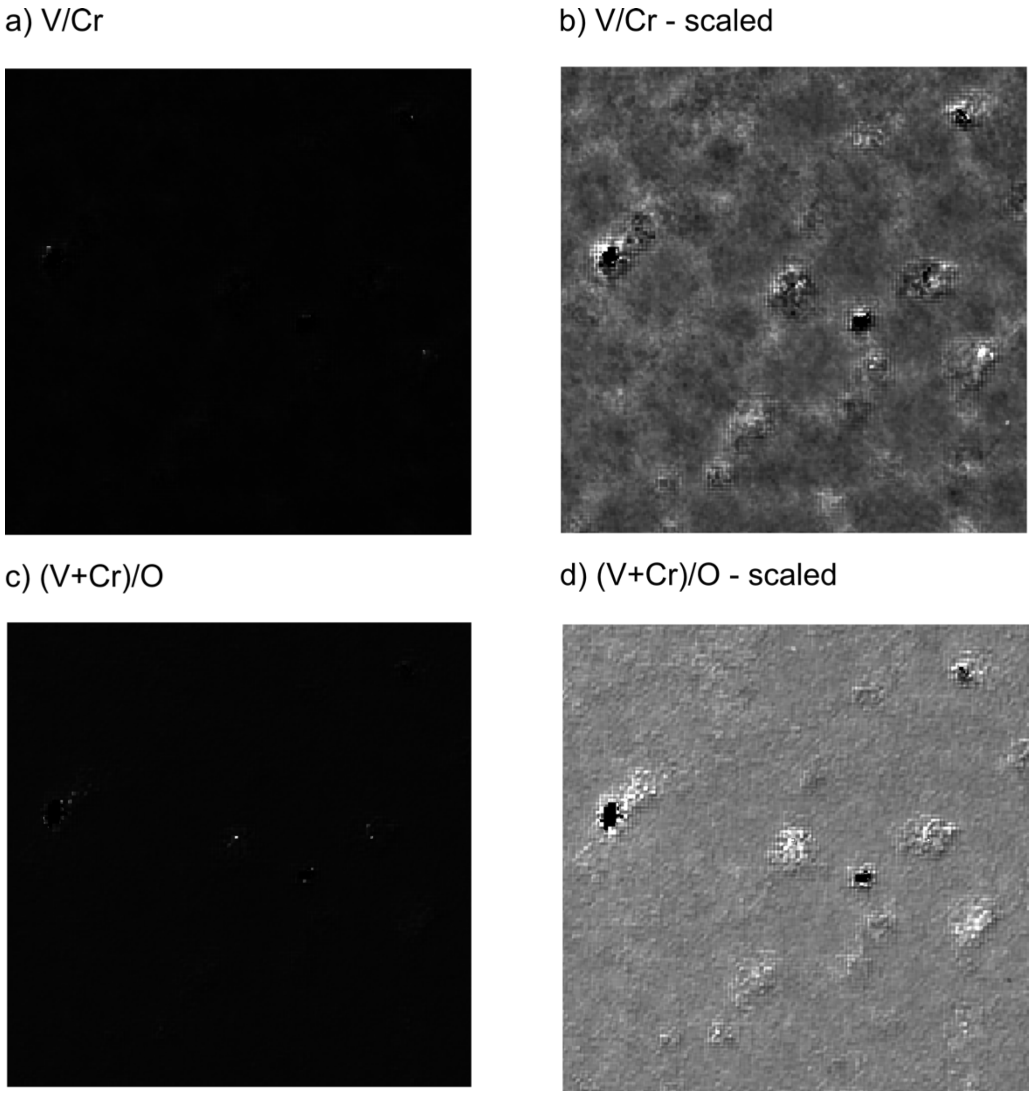

Figure S3: Ratios between the signals from Figure 4 a). The values were first scaled so that the lowest and highest brightness values match the ends of the range representable by the 8-bit image, then they were divided as indicated. a) reports the ratio between vanadium and chromium , while c) shows the sum of both in relation to oxygen. A black color indicates values close to 1, suggesting a homogeneous composition. The film does not contain vanadium and chromium in equal proportions, but a quantitative evaluation would require the measured intensity to be calibrated against a known standard. In b) and d), the results were again scaled to reveal the structure in these small values. From b) it appears like the grain boundaries are slightly vanadium rich, while in d) there is no obvious structure apart from minor fluctuations. The spots in these images most likely correspond to pores in the films.
